# How do junior doctors in the UK learn to provide end of life care: a qualitative evaluation of postgraduate education

**DOI:** 10.1186/s12904-015-0039-6

**Published:** 2015-09-23

**Authors:** Sophie Price, Susie Schofield

**Affiliations:** MacMillan Unit, Christchurch Hospital, Fairmile Road, Christchurch, Dorset, BH23 2JX UK; Centre for Medical Education, University of Dundee, Dundee, Scotland DD1 4NH UK

## Abstract

**Background:**

The fundamental importance of good end of life care has been well documented however recent national publications have high-lighted inadequacies in training in this area. For many patients dying in the UK today care is provided in hospital and the number of inpatient deaths is forecast to climb significantly in future. The demands of providing medical care for these patients by junior doctors will continue to rise. However, there is currently only limited research on training for doctors in this setting.

**Methods:**

A qualitative study using semi-structured interviews of trainees working in general medicine analysed utilising a grounded theory approach.

**Results:**

Eleven medical trainees from nine different medical schools participated. They had worked in fifteen different UK hospitals in the course of their careers. All of the doctors interviewed felt generally confident in managing a dying patient. This had developed at postgraduate level and increased when working in certain key specialties. Emerging themes fell into five main categories: perceived ability in clinical management, different learning opportunities experienced, the impact of variations in approach to end of life care, the role of the specialist palliative care team and suggestions for improvements to training. All participants felt further teaching would be beneficial.

**Conclusions:**

This study identified key areas where training could be improved. This included small changes in everyday practice to shift the emphasis for trainees to education. There also needs to be focus on end of life care in the curriculum, formal teaching programmes and assessment of junior doctors. The specialist palliative care team played a vital role in training as well as service provision. For those working in this specialty, every clinical encounter provides an opportunity for education. Specifically targeting junior doctors will not only improve patient care today but empower the consultants of the future.

**Electronic supplementary material:**

The online version of this article (doi:10.1186/s12904-015-0039-6) contains supplementary material, which is available to authorized users.

## Background

Almost 50 % of deaths in the UK occur in hospital and most will be in a generalist setting [1]. Thus almost all doctors will be involved with the management of a dying patient at some point in their career [2]. The requirement for all doctors to have the skills necessary to manage these patients is well documented – ‘Palliative care is the right of every patient, its provision the responsibility of every doctor’ Derek Doyle 1996 [3].

There has been increasing attention in recent years on the undergraduate curriculum in palliative medicine in the UK [4–7]. Despite this there is considerable evidence that newly qualified doctors feel unprepared to care for dying patients [8–10]. Many studies suggest that it is perhaps unrealistic to expect junior doctors to feel prepared for this, implying that the majority of these skills are developed at postgraduate level [9–11]. The current literature in postgraduate education for trainees concentrates on newly qualified doctors, general practitioners or specific courses [8, 12, 13]. Research into training for junior doctors in hospital posts in this area remains limited.

There have been several recent high profile national publications reviewing the care of dying patients in the National Health Service [14–16]. These have highlighted a need for improvements in education for all staff members. With an ageing population, the number of hospital deaths is forecast to increase significantly over the next 15 years [17], thus the demands of providing medical care for dying patients by junior doctors will continue to rise. It is becoming increasingly important to understand how these doctors gain the skills required, so that appropriate changes can be made to improve both education and patient care.

This study aimed to evaluate the educational experience of trainees in general medicine to identify key learning experiences and their perceived effectiveness. It also investigated the changes in confidence in managing palliative patients over the course of early medical careers. This article presents information obtained from the study, which can provide a resource for directing future education and improving training schemes.

## Methods

A qualitative approach was taken, using semi-structured interviews of junior clinicians who had experience of working in a range of hospitals throughout the UK [18]. Ethical approval was obtained for the study from the Research Ethics Committee of the University of Dundee.

### Sample selection

A sample of Core Medical Trainees (CMT) was chosen as they are the junior doctors most likely to be caring for dying patients on a regular basis [8]. CMTs have completed at least two years of generic postgraduate work and are training in general medicine before subspecialising, rotating through different medical specialties. To facilitate recruitment and interviewing a convenience sample of doctors currently working within Wessex Deanery was used. Participants were identified using Deanery records (104 in total) and recruited by e-mail invitation. Non-responders were also recruited using a snowball technique, whereby interviewees were asked if they knew other trainees who might participate [18]. Trainees were required to give written informed consent prior to participation.

### Data collection

Semi-structured face-to-face interviews were conducted by a single researcher. A topic guide was used, comprising items about end of life care including confidence in patient management, experiences in training and suggestions for improvements (Additional file 1). The interviews lasted between 25 and 70 min. Each interview was audiotaped and transcribed verbatim for analysis. All data was anonymised during transcription.

### Analytical framework and data analysis

An inductive qualitative approach was employed to derive concepts and categories from the interview data [19]. Analysis was performed by a single researcher using the constant comparison technique [20]. Codes from the text were identified and compared to those generated from subsequent interviews. As new codes emerged these were correlated with previous transcripts and emerging concepts were connected to produce themes. Saturation of themes was evident after eight interviews, but three more were performed to ensure full data saturation.

## Results

### Participant demographics

The 11 doctors who were interviewed had qualified from nine different medical schools and worked in 15 different hospitals throughout the UK. Three had also taken time out of training to work abroad. Their length of experience in postgraduate training ranged from two years and six months to five years and nine months, (average three years and eight months). There were nine female and two male participants. There was a broad range of career aspirations including Cardiology, Gastroenterology, General Practice, Medicine for Older People, Oncology, Palliative Care and Rheumatology. One trainee was undecided at the time of interview.

The overarching theme emerging from the data was the different learning opportunities encountered during medical training and the influence this had on clinical confidence. Emerging sub-themes fell into five main categories: perceived ability in clinical management, different learning opportunities experienced, the impact of variations in approach to end of life care, the role of the specialist palliative care team (SPCT) and suggestions for improvements to training.

### Perceived ability in clinical management

All the participants stated they felt generally confident in managing a dying patient. For most this confidence had developed in postgraduate work, over a period of years.

This perceived competence however did vary in different aspects of palliative care. Most felt comfortable with basic symptom control and communication skills, but some still described uncertainty with identifying a dying patient and prognostication.

*‘I find one of the most difficult things for me is when you are not sure about prognosis and I know that you can never be one hundred percent sure, but in some situations it is more uncertain. So especially for example, like end stage heart failure, it can be really difficult and I find that really hard, because I don’t know how to help the family, because it’s obviously hard for them.’*

#### Coping with working independently

It was clear from all the interviews that confidence increased with experience and exposure to patients with a terminal illness. As first year doctors however, many expressed a daunting feeling that they were often the one “being left” to care for these patients.

In particular a theme emerged about a fear of making things worse for the patients or relatives as a result of inexperience.

*‘I think most F1s* [Foundation Year One doctors] *just feel “if I give too much of this, this could cause a problem”, so they don’t give anything……they don’t give anything and they’re in pain, but they’re scared to give morphine because they’re worried it might………stop them breathing or something.’*

The doctors’ perceptions of the senior support available in these situations varied considerably. Whilst some felt that a dying patient was a high priority for their seniors, others were much less positive:

*‘I would go to senior colleagues ….. who were very dismissive about it and didn’t seem to…… not ‘didn’t seem to care’, but I don’t think that they knew what to do about it either.’*

### Different learning opportunities

The interviewees described five key learning opportunities: formal teaching sessions, informal teaching, experiential learning, observation of colleagues and written guidance.

#### Formal teaching sessions

The amount of dedicated postgraduate teaching described was very variable. Some reported little or even no formal teaching since medical school.

Formal sessions were however often part of the undergraduate programme, although perceptions of its value varied considerably. Whilst some were very positive about medical school teaching, it was often acknowledged it had not prepared them for clinical practice.

*‘[In medical school] I definitely saw a lot ..….. about symptoms and things, but never really got taught, kind of, how I would manage it when I became a doctor………..… obviously it’s good to have any exposure to palliative care, but I don’t think it was particularly useful for my training, in terms of “now you need to be able to manage that yourself”.’*

The absence of formal teaching for CMT grade was mentioned in many of the interviews. Seven of the doctors reported no specific teaching on end of life care and the others described minimal teaching only. Various reasons for this were suggested including low priority in the curriculum and the practicalities of didactic teaching on this subject. Participants did report emphasis on communication skills training however, often driven by assessment requirements for postgraduate examinations.

#### Informal teaching

Informal teaching on the ward and discussions with other staff were a valuable learning opportunity. This came principally from the SPCT, but informal teaching on ward rounds from non-specialist teams was also important. Ward nurses were mentioned as a source of guidance both for prescribing and communication with relatives. One doctor also described using case-based discussions around end of life care (part of the formal assessment process for all trainees) as a particularly useful learning tool.

#### Experiential learning

All of the doctors described ‘learning by doing’ and that ‘real life experience’ improved confidence the most. Some were able to describe more specifically their reflective process of learning from individual patients although almost all were situations the doctors felt could have been better managed.

*‘“…a patient who was clearly dying and the surgeon just wouldn’t accept to withdraw treatment and it was just not a nice experience …… when this guy was clearly at the end of his life. I felt that was an experience that has kind of stuck with me and that was 5 years ago and it has, I guess, made me reflect and think if I get to that point with other patients to, kind of, recognise when is the time to say “that’s enough, keep them comfortable”.’*

#### Learning from observation

Observing colleagues emerged as the principal opportunity for improving communication skills. Participants often described how they adapted their own communication styles after watching consultants relay difficult news.

*‘It’s just nice to see how someone else addresses and uses a phrase that you might not have thought of, but actually that is a very good way of putting it - yes I could use that again.’*

Unfortunately observing colleagues was not always a positive experience. Many recounted episodes of poor practice, but were, at least, able to identify ‘what not to do’ from these experiences.

#### Written guidance

Almost all of the doctors interviewed discussed written guidance in various forms. In addition to booklets and handbooks, all but one mentioned specific end of life care plans as a learning tool, in particular for prescribing. Some also discussed medication ‘bundles’ available on electronic prescribing systems that guided their treatment choices.

#### Missed opportunities for teaching

All of the doctors reported areas where teaching was deficient, which often correlated with areas in which they felt less confident, for example titrating syringe drivers. In addition to the absence of formal teaching for CMTs, a lack of focus on palliative care in general for this grade emerged from the interviews. Suggested reasons included the CMT curriculum and organisation of rotations.

*‘In my core training years………I think the main focus has been on service provision and the opportunities for training, particularly in palliative care - there haven’t been that many’*

### The impact of variations in approach to end of life care

All interviewees reported considerable variation in approach to end of life care in different posts that impacted on training. Exposure to dying patients was a significant factor in this. Key specialties were identified in which participants gained more confidence and were often given more teaching. These included oncology, elderly care and respiratory medicine.

The doctors also described posts where teams had a different approach, usually in specialties where a patient death was a less frequent occurrence. Focus here was on active management and issues around end of life care often not considered.

*‘Earlier this year I did cardiology and …… there is definitely a different mind-set. Very few patients have any sort of advanced decisions made. It’s quite interventional and quite ……. aggressive in management’.*

The most notable differences were around failures in recognising a dying patient and lack of clarity around treatment plans. There were several discussions as to why this should be, including the personal views and past experiences of the consultants, as well as the general ethos of the specialty.

*‘They’re not supposed to die, are they - because they’re surgical and that ruins their lists. They’re not allowed to die on surgery!’*

### The role of the specialist palliative care team

Regardless of specialty, the SPCT was the biggest single source of teaching both formal and informal.

*‘I’ve just kind of picked things up from Palliative care nurses that have come to see the patient……Just from talking to them and asking them question. Asking them why they had chosen that particular medication and talking to the palliative care consultants out of hours.’*

Working patterns and workload sometimes prevented direct discussion with the team, although ‘indirect’ learning was still felt to be possible from comments in the notes.

A specific theme regarding the role of the SPCT in general emerged from almost all of the interviews. The advice given by the SPCT was always welcomed by the participants:

*‘I think they are one of the teams that come and they kind of “save the day!” …. it is always reassuring when the palliative care team have been.’*

However, following referral the role of the original clinical team was very variable. Most of the participants perceived that there was often considerable reliance on the SPCT, which could be avoided with better education.

*‘I feel there is a bit of …… “Oh, refer palliative care”. There’s no plan, it’s just refer [to] palliative care ….. and I feel that we de-skill ourselves’*

Whilst this reliance provided consistency of care, particularly in specialties with less exposure to palliative care, the trainees recognised they still needed to be able to manage these patients themselves.

*‘We did have a lot of support from the palliative care team, but there is only so much that they can do, they can’t be there the whole time……..You can’t expect the palliative care team to hold your hand the whole way’*

### Suggestions for improvements to training

All those interviewed felt further education in end of life care would be useful. There was a wide variety of suggestions for the best format, including didactic lectures, small group work and additional written guidance. All agreed teaching needed to be more clinically orientated.

*‘I tend to find that most of the teaching we have has never addressed what it is like on the ward……So I think a lecture that addresses a case of a patient and how you manage it step by step…… So that when I’m on the ward, I can be confronted with a similar situation then obviously realise “oh yes, I learnt that in this lecture last week”.’*

Joint consultations with the palliative care team were felt to be an underused resource and an ideal opportunity for case-based learning, although workload and time pressures were often reported as a barrier.

The absence of formal teaching sessions and the low profile of palliative medicine for CMTs was a common theme. However, often the trainees felt that the focus should be on developing teaching for newly qualified doctors:

*‘I think it would be useful, particularly for the more junior doctors for more support from the hospital palliative care team in that respect……helping the first years. Helping with how to discuss dying with the patient’s family, how to discuss resuscitation decisions and basic symptom management.’*

## Discussion

These accounts explore the experiences of junior doctors as they learned to care for people who were dying. At the time of interview they all felt generally confident in the essential elements of end of life care and this had developed over several years, mostly in postgraduate education.

### Confidence as a foundation year doctor

In line with related literature, the majority acknowledged that their medical school education on palliative care had not been an adequate preparation for work as a doctor [8, 10]. For many, undergraduate teaching had provided a general overview, but not the tools necessary to manage a patient on their own. The feeling of ‘being left’ to care for dying patients is reflected in other literature [10], but in addition, participants described a fear of making things worse for the patients or families due to inexperience. Whilst there is some literature suggesting junior doctors are responsible for a significant number of prescribing errors [21], it is difficult to know if these concerns are justifiable. Interestingly in one study of prescribing by first year doctors [21], analgesia was an area where they felt more confident. This contrasts with the views of some of the participants here, perhaps reflecting anxieties about the specific needs of a dying patient.

### Learning in different specialties

Descriptions of the senior support available for the doctors were variable and often specialty dependent. As with other studies [8, 22], differences in individual doctor and specialty approach had a substantial impact on education. Experience in specialties with more exposure to life-limiting illnesses, in particular oncology, medicine for older people and respiratory medicine, was a significant factor in improving confidence. This reflects the guidance in the Department of Health End of Life Care Strategy, [23] which stratifies staff into three groups dependent on the frequency of such interactions, suggesting different levels of skills were appropriate for each. However it acknowledged that all healthcare professionals should still have a “good basic grounding in the principles and practice of end of life care” and know when to seek specialist advice.

### The specialist palliative care team

Consistent with literature about newly qualified doctors [8], the SPCT was unanimously cited as an invaluable source of advice in end of life care, especially in specialties with limited teaching from senior colleagues.

The SPCT was discussed in all interviews, with regard to both education and its more general role. There was an impression from the majority of participants that once a decision had been made to take a palliative approach, all further decisions about care were often left to this team. With better education it was suggested there could be less reliance on the specialist team. Gott et al. [24] explored a similar theme in research on healthcare staff’s perspectives of the remit of palliative care. This considered how the evolution of specialist palliative care as a discipline has altered the involvement of other health care providers in this aspect of care. It was suggested that the SPCT may need to focus on ‘empowering’ other health care professionals rather than ‘taking over’.

The impact of the SPCT in education was one of the most striking findings from the data and demonstrates that every clinical encounter is a potential opportunity for teaching. Unfortunately this was sometimes limited by workload and working patterns. This key role in education is acknowledged in several national documents [14, 25] highlighting this is an integral part of clinical service which must be considered when commissioning and planning services.

### Different learning experiences

Five key areas emerged as important for developing clinical confidence – formal teaching sessions, informal teaching, experiential learning, observation of colleagues and written guidance. These educational methods correlate with literature on other groups of doctors [22, 26]. One such American study [26] discussed ‘learning from others’, where participants were unanimously positive about advice from senior doctors. In contrast, the current British participants felt there were some circumstances when they were learning “what not to do”. They recognised that they were using the identification of poor practice as a learning experience in itself. This reflects national concerns about the quality of training and education that all doctors receive in end of life care [14].

Learning from written guidance is not generally a feature of the other literature on education in palliative care, although one paper demonstrates the benefit of a ‘pocket-card’ prompter in symptom control [27]. This may reflect the use of a regional palliative care handbook [28], as well as recent changes in documentation around end of life care [14, 15]. The benefit of written guidance for prescribing was particularly emphasised in the current interviews, with the trainees recognising the role of patient care plans in education, as well as hands-on care.

### Suggestions for improvements to training

All of the doctors agreed that further training would be beneficial and more formal teaching, written guidance and further opportunities for observing the SPCT were most commonly suggested. The overall consensus that teaching needed to be more case-based is consistent with adult learning theories [29, 30] and emphasises the importance of linking education with the experiences of everyday life, which many trainees felt was currently neglected.

It is generally accepted that assessment often drives learning [31] and certainly some of the formal communication skills training centred around postgraduate examinations. However, overall it was felt that palliative medicine had a low profile for Core Trainees. This may well be a regional effect, as the participants’ Core training was all within one Deanery, although their formal teaching programmes were independently designed by their trusts. One participant suggested it might be a reflection of the content of the core curriculum. A recent national report [15] discussing approaches to care of the dying patient reviewed the curricula of trainees. It identified several curriculum items relating to end of life care, however these were mostly generic professional competencies. This report also highlighted training resources available, many of which were e-learning and on-line documents. In contrast the learning methods used by the participants in the current study were almost exclusively workplace based, rather than additional courses.

### Limitations

As with many qualitative studies, these findings rely on the individuals’ perceptions and confidence rather than a measure of clinical care. The study was designed to explore the learning experiences of the doctors, but the qualitative approach relies not only on a memory of events, but also the participants’ perception of what constitutes learning. For example, one doctor discusses ‘advice’, differentiating this from teaching. Similar experiences may not have been reported by other participants, who may not have recognised the educational value of the encounter.

Participants were recruited through both an e-mail request and a snowballing technique. This aimed to capture a range of trainees, rather than just those with a particular interest in palliative care. There is no data available on those who did not respond to the request or their reasons for this. Although some participants did express more confidence than their colleagues as a result of a special interest, there was little difference in the themes emerging from these interviews. Despite small numbers for the study, there was saturation of themes after eight interviews.

Core Medical Trainees were chosen for the study, as they were felt to be those most likely to work with dying patients. Although the undergraduate and Foundation Year training, which formed the majority of the experiences described by the participants, is generic for all doctors, their more recent training is likely to be significantly different from that of other specialties. The learning needs of those less commonly involved in the care of the dying patients have not been explored in this study and would be an important aspect for future research. In addition, the participants’ choice of a career in medicine is likely to have significantly influenced their perceptions and descriptions of other specialties. Although generic training was undertaken in a wide range of UK hospitals, the participants’ Core Medical training was all undertaken in Wessex Deanery, so discussion of this aspect may not be more widely generalisable.

Due to limitations in resources, interviews and data analysis was carried out by a single researcher undertaking specialist training in palliative care. The researcher worked in the same locality as the participants, with the potential for future clinical encounters. Therefore the researcher’s perceived role as a ‘senior expert’ in Palliative Care may have influenced the responses of the interviewees, particularly in discussions about the SPCT. To try to minimise this effect it was made clear to interviewees that this was about personal experience, with no ‘right or wrong answer’.

## Conclusions and implications

This study describes how junior doctors’ confidence in end of life care increased as their careers progressed and the learning experiences that influenced this. The concepts discussed have significant implications for improving education, often by simply recognising the learning potential of everyday activities and shifting the focus to education. This impacts not only specialists in palliative care, but those in other secondary care specialties, curriculum planners and policy makers. Simply observing consultant interactions with relatives was shown to be a valuable opportunity for informal teaching. Taking just a few minutes to reflect on learning outcomes with junior team members is an easy way to achieve the often challenging task of incorporating education into daily work. Similarly, encouraging examples such as work-place based assessments around end of life care provides a formative as well as a summative exercise. There needs to be ongoing focus on palliative medicine in the curriculum and assessment of medical trainees. The core teaching programme for junior doctors is often linked to curriculum requirements and provides an ideal platform for more formal teaching. This teaching needs to be clinically orientated and explicitly linked to the experiences that trainees will encounter on the wards.

The results also emphasise the role that the everyday activities of the SPCT play in training and that this is inseparable from service provision. Every interaction with a patient or trainee is an opportunity for teaching. This is especially important for teams with limited exposure to palliative care, providing both consistency of care and education. Encouraging junior doctors to join the specialist team for patient reviews can provide the case-based, clinically relevant approach to teaching requested.

There is increasing evidence of the need for improvements in training in end of life care [14]. An aging population and predicted rise in the number of in-hospital deaths will have a significant impact on the work of medical trainees. Increasing both formal and informal teaching opportunities can help to embed the principles of good quality care into practice. Specifically targeting the junior doctors will not only improve patient care today, but empower the consultants of the future.

## Additional file

Additional file 1:
**Outline of interview schedule and examples of questions and probes.** (PDF 105 kb)

## Abbreviations

CMT: Core Medical Trainee
F1: Foundation Year One Trainee
SPCT: Specialist Palliative Care Team
UK: United Kingdom
