# Supplementary material for: How do junior doctors in the UK learn to provide end of life care: a qualitative evaluation of postgraduate education
Source: BMC Palliat Care. 2015 Sep 23;14:45. doi: 10.1186/s12904-015-0039-6 (PMC4580223; doi:10.1186/s12904-015-0039-6)
Supplement: Additional file 1: — Outline of interview schedule and examples of questions and probes. (PDF 105 kb) [file 12904_2015_39_MOESM1_ESM.pdf]

Additional file 1: Outline of interview schedule and examples of questions and probes

Focus area	Examples of questions and probes
Demographics	What year of training are you in?<br>Where are you currently working?<br>Where else have you worked?<br>Which Medical school did you attend?<br>What field of medicine do you hope to work in?
Confidence in patient management	How confident do you feel in managing a patient who might be approaching the end of their life?<br>Has your training so far has enabled you to cope with your encounters with dying patients?
Experiences in training	How do you think that you learned to care for patients who are dying?<br>What experiences have you found most helpful in learning how to manage patients who are thought to be dying?<br>Why was this useful?<br>In the last few years, what teaching have you had on end of life care?<br>What type of teaching was that?<br>Have you had training other than formal teaching sessions?<br>Who has provided the most education in this area?<br>What do you think were the most useful learning opportunities?
Suggestions for improvements	What could have been improved about your training?<br>Would you like more teaching in Palliative or end of life care?<br>What would be most helpful for you?
Close	Is there anything else you would like to add that has not already been discussed?<br>Do you have any comments or concerns about what we have discussed?
